# Supplementary material for: Telomere Dysfunction in Human Astrocytes Drives Acrocentric Chromosome Instability and Nucleolar Reorganization
Source: bioRxiv. 2026 May 21:2026.05.19.726354. Preprint. [Version 1] doi: 10.64898/2026.05.19.726354 (PMC13228289; doi:10.64898/2026.05.19.726354)
Supplement: Supplement 1 [file NIHPP2026.05.19.726354v1-supplement-1.pdf]

bioRxiv preprint doi: https://doi.org/10.64898/2026.05.19.726354; this version posted May 21, 2026. The copyright holder for this preprint (which was not certified by peer review) is the author/funder, who has granted bioRxiv a license to display the preprint in perpetuity. It is made available under a CC-BY 4.0 International license.

Supplementary Figures

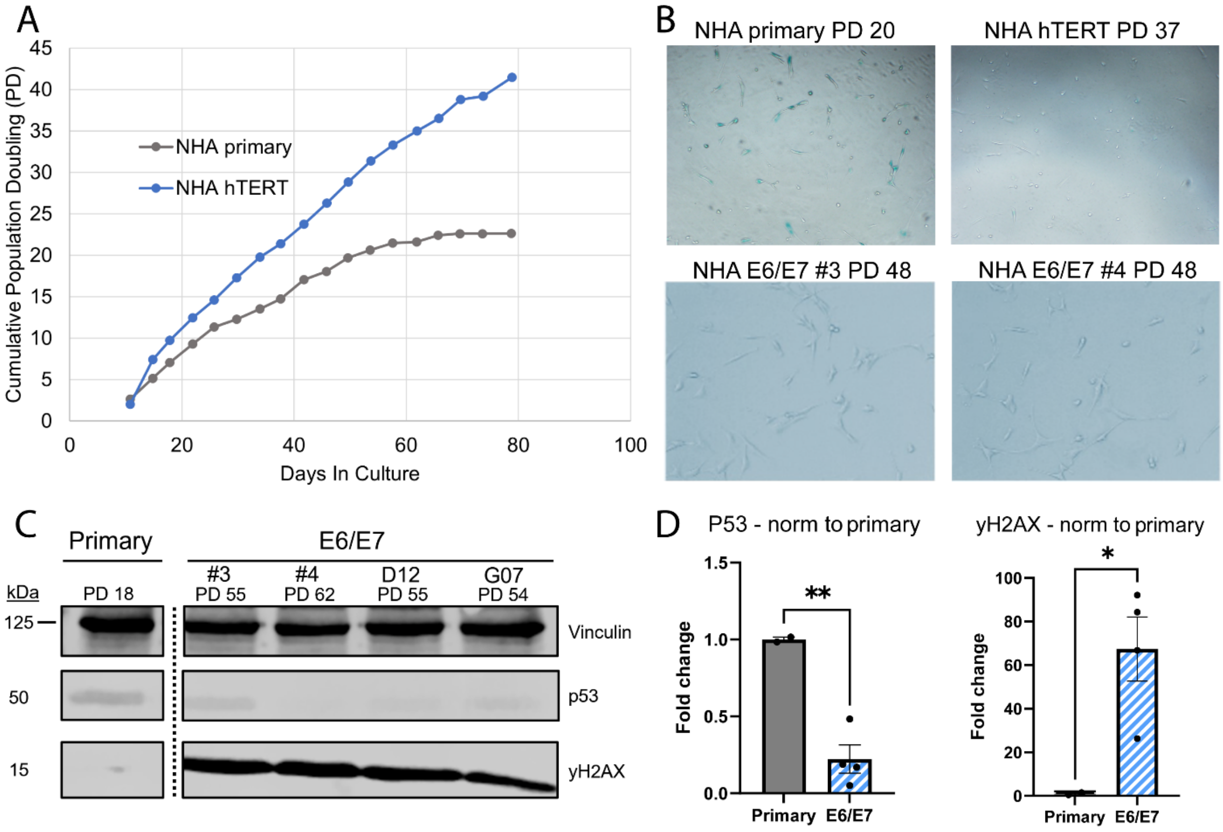

Supplemental Figure 1: E6/E7 model survives past primary senescence points while DNA damage markers increase significantly. **(A)** Growth curves of NHA primary vs NHA hTERT. The population doubling of the primary line plateaus at PD22, while hTERT continues to grow past PD 40 at day 80. **(B)** Beta-galactosidase staining of NHA primary, hTERT, clone #3, and #4 to assess senescence. Primary NHA cells began to senesce at PD 20 as indicated by blue staining within individual cells, while NHA hTERT and the E6/E7 transduced clones show no signs of senescence **(C)** Western blot of NHA primary vs E6/E7 measuring p53 levels and yH2AX levels at late passages. **(D)** Quantification of western blot data confirms significantly low levels of p53 and high levels of yH2AX in primary vs E6/E7.

bioRxiv preprint doi: https://doi.org/10.64898/2026.05.19.726354; this version posted May 21, 2026. The copyright holder for this preprint (which was not certified by peer review) is the author/funder, who has granted bioRxiv a license to display the preprint in perpetuity. It is made available under a CC-BY 4.0 International license.

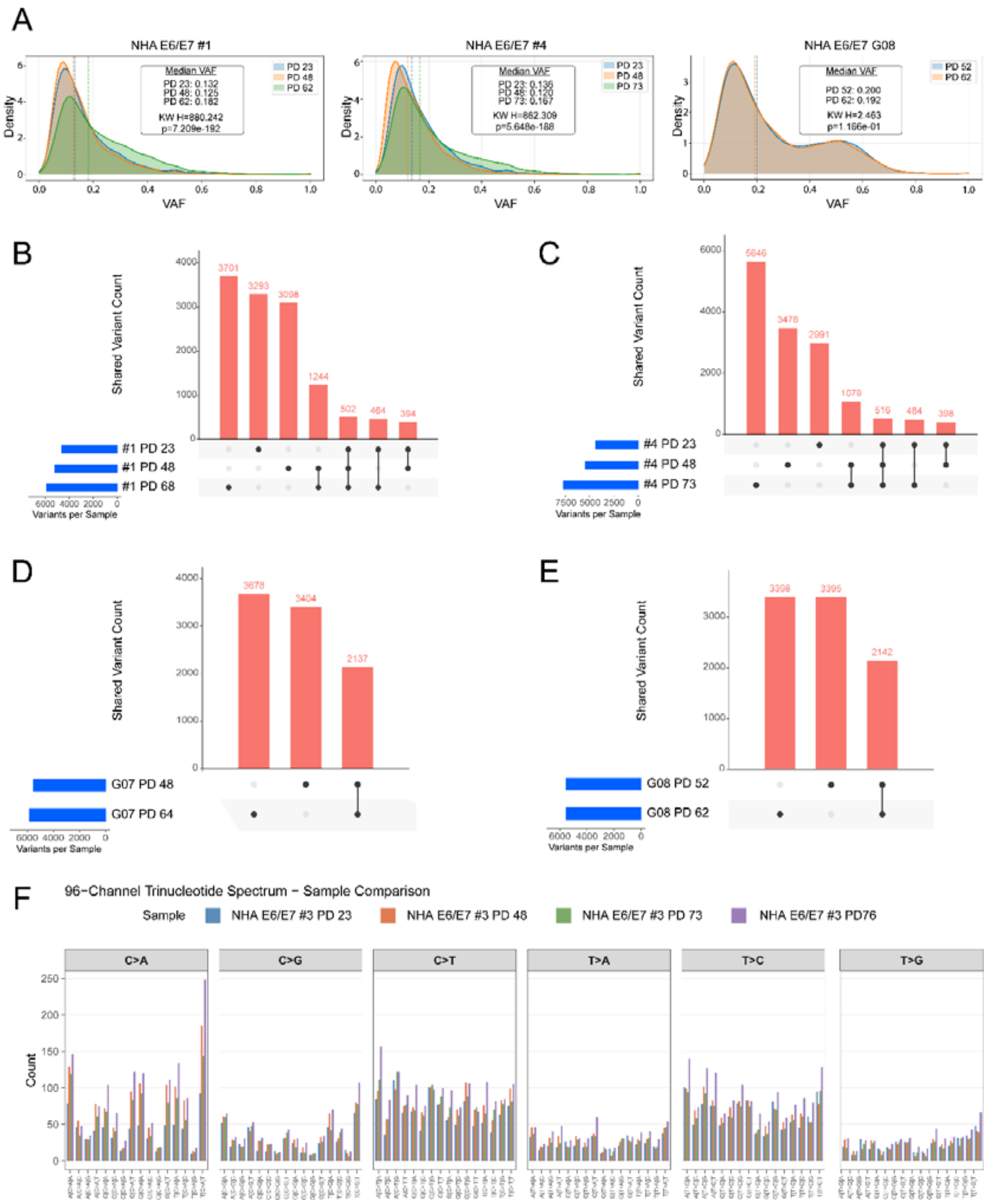

Supplemental Figure 2: Additional VAF distribution data and upset plots for remaining clones. (A) Variant allele frequency (VAF) density distributions for representative samples (bulk #1, #4, and single-cell lone G08) at progressive PDs. Dotted vertical lines denote median VAFs, with Kruskal-Wallis (KW) test statistics provided for each clone. (B-E) UpSet plots illustrating the intersection of shared and unique SNVs and indels across multiple PDs for bulk clone #1 **(B)**, #3 **(C)**, and single-cell clones G07 **(D)** and G08 **(E)**. **(F)** The 96-channel single-nucleotide substitution (SNS) spectrum is shown for bulk clone #3 at PD 23, 48, 73, and 76. Mutations are grouped by substitution type: C>A, C>G, C>T, T>A, T>C, and T>G, and further subdivided by 5' and 3' flanking nucleotides. The Y-axis indicates raw mutation count. Colors denote individual passage time points.

bioRxiv preprint doi: https://doi.org/10.64898/2026.05.19.726354; this version posted May 21, 2026. The copyright holder for this preprint (which was not certified by peer review) is the author/funder, who has granted bioRxiv a license to display the preprint in perpetuity. It is made available under a CC-BY 4.0 International license.

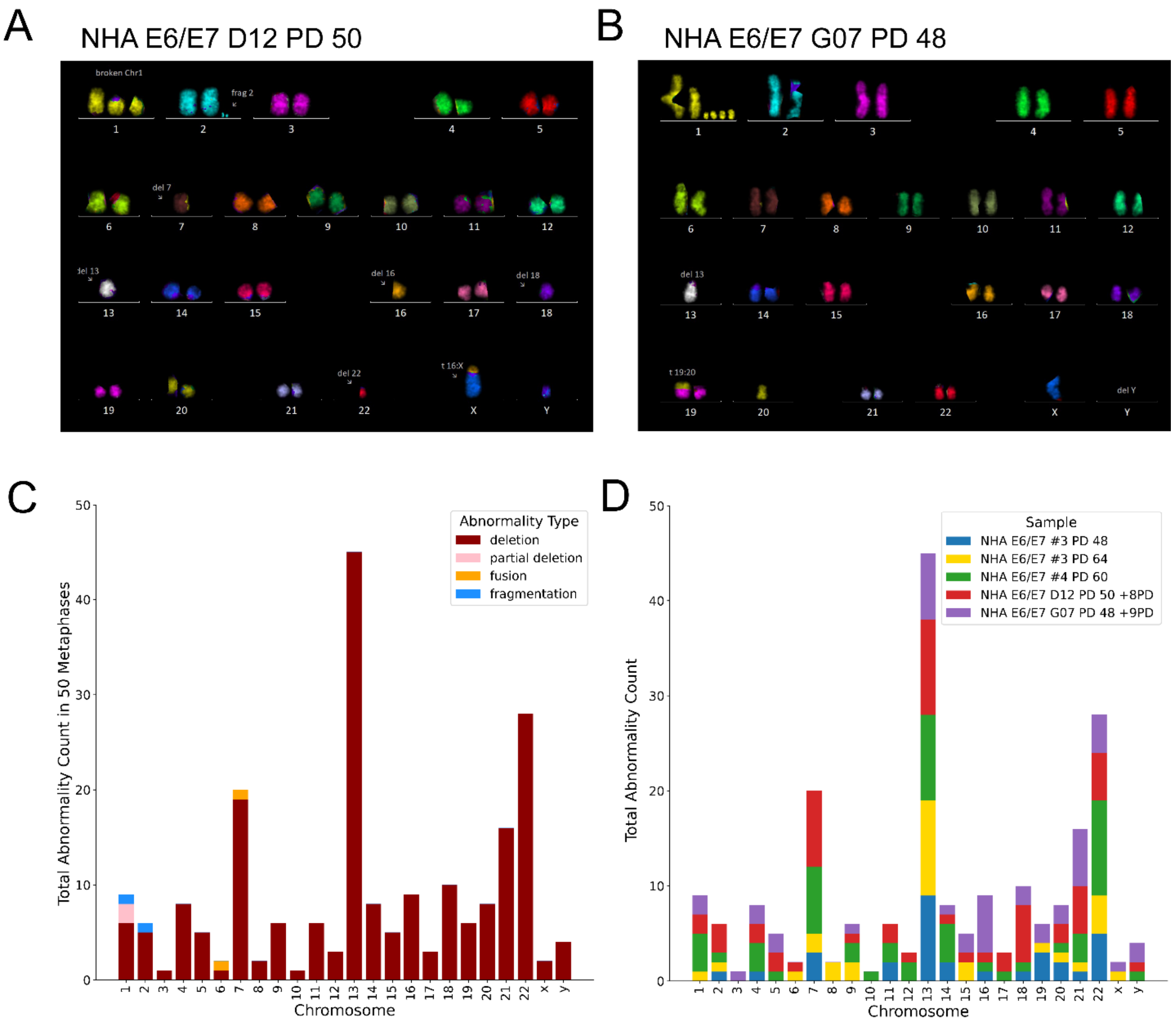

Supplemental Figure 3: 24-color mFISH for additional NHA E6/E7 clones. (A) Clone D12 shows chromosome 7,13, 16, 18, and 22 deletion, and translocation of chromosome 16 to chromosome X. (**B)** Clone G07 showing fragmentation of chromosome 1, deletion of chromosome 13, 20, & Y, and translocation of chromosomes 19 with a residual segment of 20. (**C)** Abnormalities per chromosome broken down by four major types. Deletions were predominantly seen across all chromosomes. Partial deletions, fusions, and fragmentations occurred at roughly equal counts (**D)** Count of abnormalities seen within each chromosome across specific clones. Abnormality counts were not equally proportional in all samples.

bioRxiv preprint doi: https://doi.org/10.64898/2026.05.19.726354; this version posted May 21, 2026. The copyright holder for this preprint (which was not certified by peer review) is the author/funder, who has granted bioRxiv a license to display the preprint in perpetuity. It is made available under a CC-BY 4.0 International license.

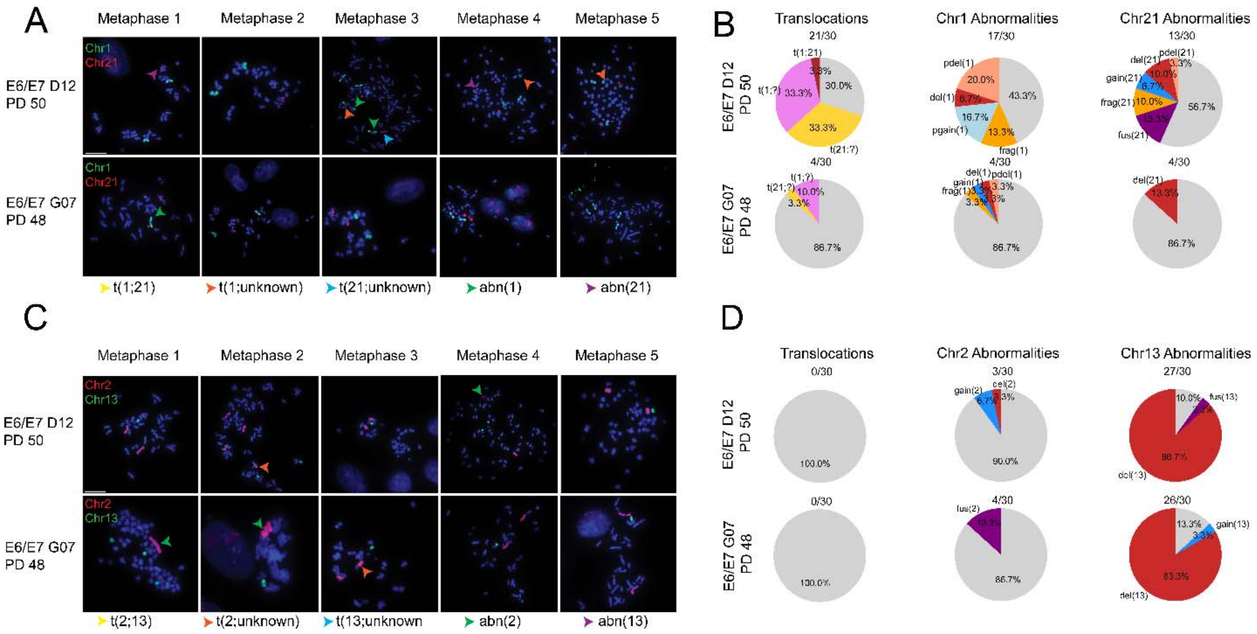

Supplemental Figure 4: Additional CT images for D12 and G07. (A) Clone D12 and G07 metaphases hybridized with CT1 and CT21. Arrows denote abnormality types seen within each cell, with yellow arrows marking t(1;21), orange marking t(1;unknown), blue marking t(21;unknown), green marking chromosome 1 abnormalities, and purple marking chromosome 21 abnormalities (**B)** Analysis of translocations and abnormalities observed in chromosome 1 and 21. Translocations involving either chromosome are noted. Those involving chromosomes 1 or 21 and unknown chromosomes were seen at 33.3% each. Those involving chromosomes 1 and 21 specifically were only seen in 3.3% of cells (n=30). **(C)** Clone D12 and G07 metaphases hybridized with CT2 and CT13. Arrows denote abnormality types seen within each cell, with yellow arrows marking t(2;13), orange marking t(2;unknown), blue marking t(13;unknown), green marking chromosome 2 abnormalities, and purple marking chromosome 13 abnormalities **(D)** analysis of translocations and abnormalities observed in chromosome 2 and 13. No translocations were seen for either chromosome, but deletion of chromosome 13 became prevalent. Fusions appeared in both single cells clones at low rates.

bioRxiv preprint doi: https://doi.org/10.64898/2026.05.19.726354; this version posted May 21, 2026. The copyright holder for this preprint (which was not certified by peer review) is the author/funder, who has granted bioRxiv a license to display the preprint in perpetuity. It is made available under a CC-BY 4.0 International license.

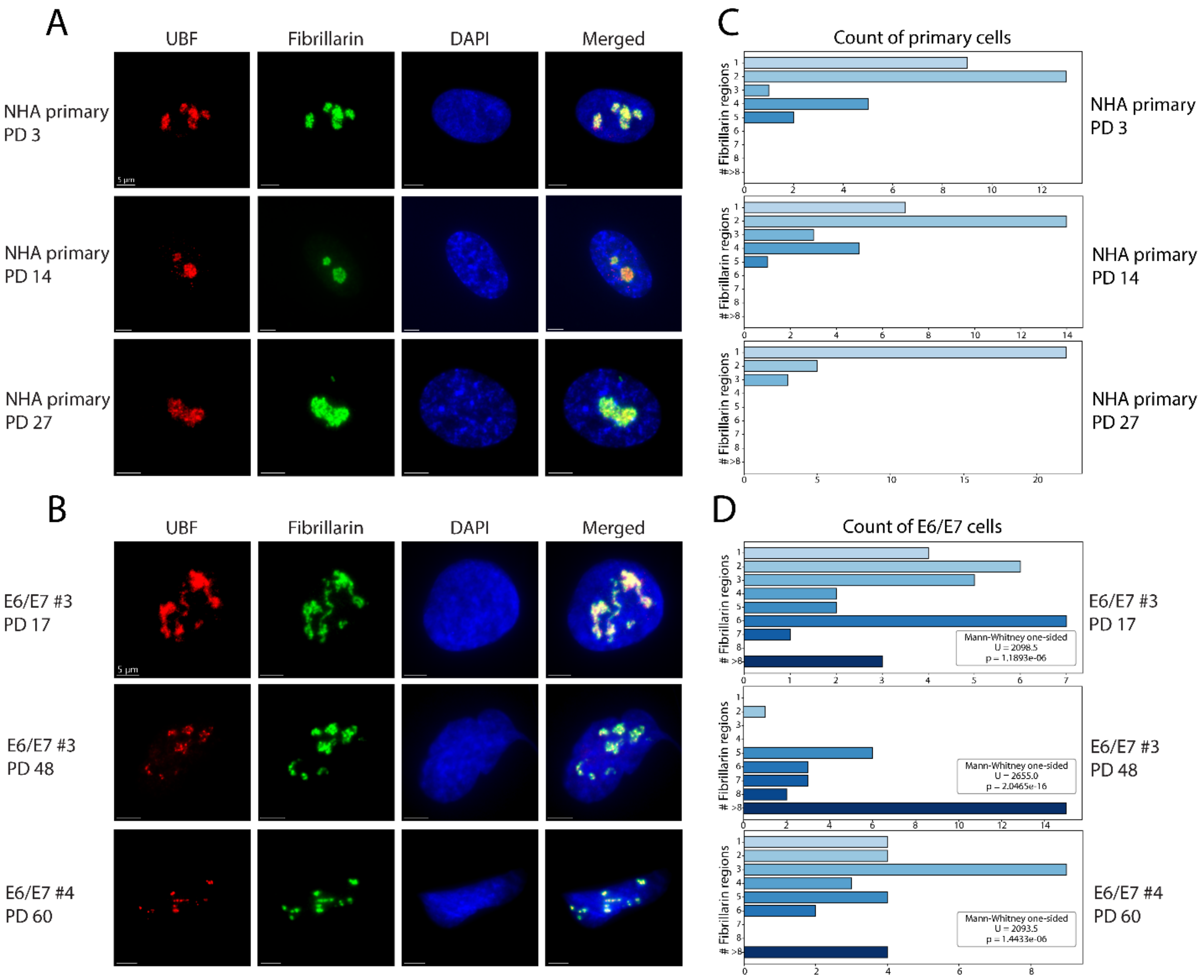

Supplementary Figure 5: Telomere dysfunction disrupts nucleolar organization in NHA E6/E7 cells. (A) NHA primary cells immunostained with fibrillarin (green) and UBF (red) antibodies at three different PDs representing young, middle, and old timepoints (PD 3, 14, and 27 respectively). Primary cells show distinct spherical boundaries denoting the nucleoli **(B)** E6/E7 cells were similarly stained with fibrillarin and UBF at different timepoints (PD 17, 48, and 60). The spherical boundaries unraveled into more linear structures that covered a wider area of the nucleus. **(C)** Number of distinguishable fibrillarin regions in NHA primary cells at each time point n=30 per time point. **(D)** Number of distinguishable fibrillarin regions in E6/E7 cells at each time point n=30 per time point.

bioRxiv preprint doi: https://doi.org/10.64898/2026.05.19.726354; this version posted May 21, 2026. The copyright holder for this preprint (which was not certified by peer review) is the author/funder, who has granted bioRxiv a license to display the preprint in perpetuity. It is made available under a CC-BY 4.0 International license.

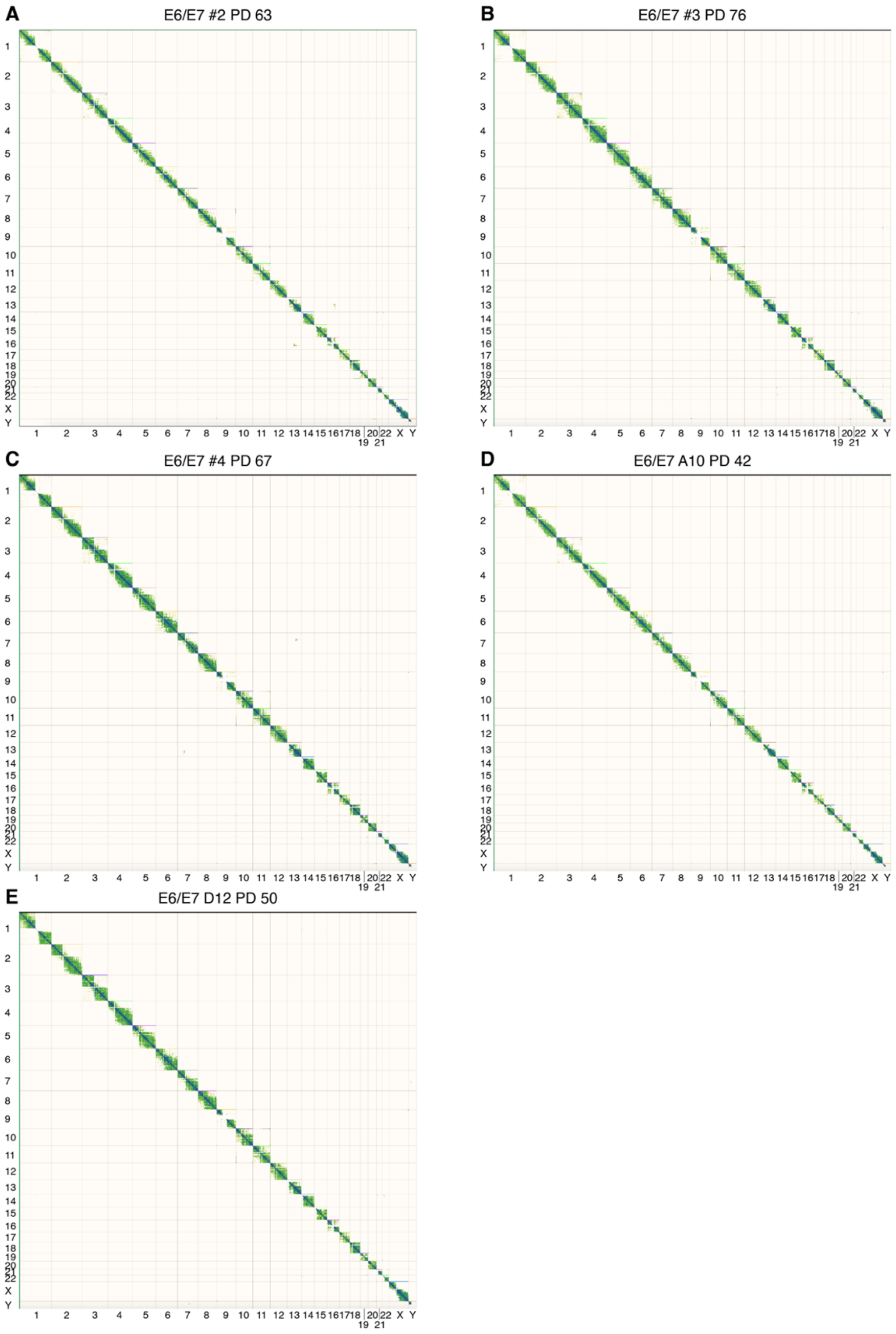

Supplementary Figure 6: Standard Hi-C contact maps for additional NHA E6/E7 samples. Genome-wide alignment-based Hi-C contact maps for NHA E6/E7 #2 PD 63 **(A)**, NHA E6/E7 #3 PD 76 **(B)**, NHA E6/E7 #4 PD 67 **(C)**, NHA E6/E7 A10 PD 42 **(D)** and NHA E6/E7 D12 PD 50 **(E)**.

bioRxiv preprint doi: https://doi.org/10.64898/2026.05.19.726354; this version posted May 21, 2026. The copyright holder for this preprint (which was not certified by peer review) is the author/funder, who has granted bioRxiv a license to display the preprint in perpetuity. It is made available under a CC-BY 4.0 International license.

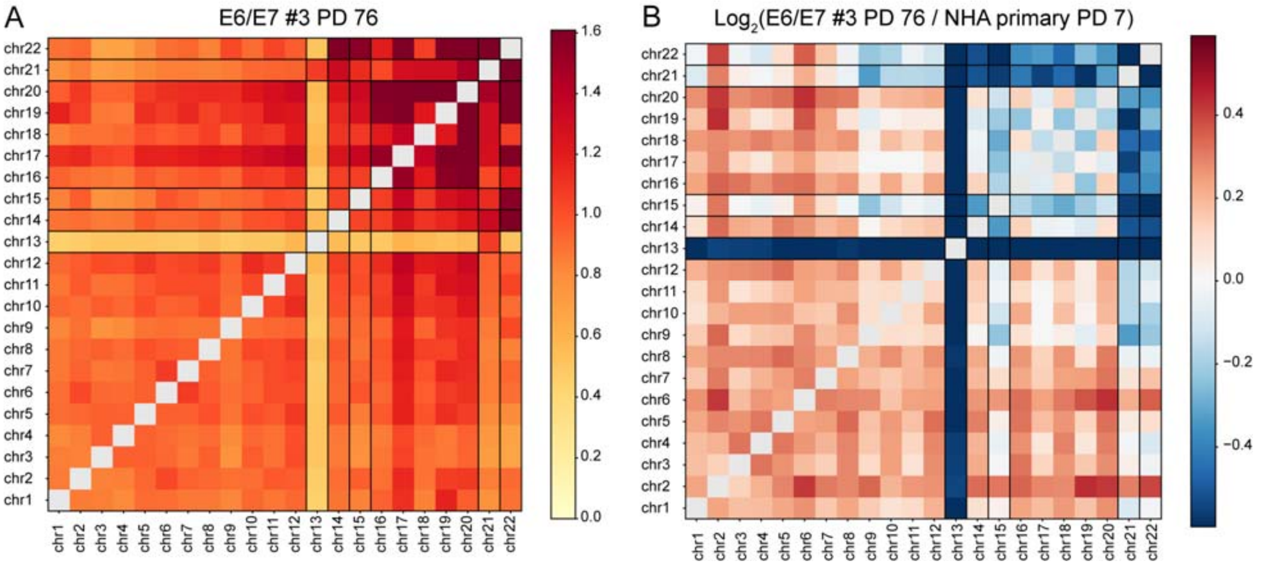

Supplementary Figure 7: KaryoScope analysis of NHA E6/E7 #3 PD 76. (A) KaryoScope-derived inter-chromosomal contact map for NHA E6/E7 #3 PD 76, displayed as observed/expected (O/E) normalized contact frequencies. **(B)** Log_2FC ratio of O/E-normalized inter-chromosomal contact frequencies between NHA E6/E7 #3 PD 76 and NHA primary PD 7. Blue indicates contact depletion in E6/E7 cells, red indicates enrichment. The chromosome 13 depletion signature and altered acrocentric contacts observed at PD 48 (Figure 5E) persist at PD 76, indicating that the nucleolar reorganization phenotype is maintained across passages.
